# Red Meat, Renal Function, Race and Other Predictors of Circulating Nitrates

**Published:** 2019-02-01

**Authors:** Samantha Sonoda, Edward Giovannucci, Kelly Hagadorn, Intan Purnajo, Bruce Bracken, Hongxia Liu, Tianying Wu

**Affiliations:** 1 Cincinnati Children’s Hospital, Cincinnati, USA; 2 Departments of Nutrition and Epidemiology, Harvard School of Public Health, USA; 3 Division of Epidemiology and Biostatistics, School of Public Health, San Diego State University, USA; 4 Department of Surgical Urology, University of Cincinnati College of Medicine, USA; 5 Beijing University of Chinese Medicine, China

**Keywords:** Urinary nitrate, Plasma nitrate, Diet and lifestyle, Biomarker, Red meat, White meat

## Abstract

**Introduction:**

Increased levels of circulating nitrates were associated with both beneficial and adverse health outcomes in observational and intervention studies. Although more experimental studies are needed to study the underlying mechanisms, it is important to determine the association between multiple dietary and lifestyle factors and circulating nitrates in observation studies to help understand the complicated relationships between circulating nitrates and disease outcomes.

**Objective:**

We plan to determine the associations of red meat, renal function, race, gender, and other lifestyle factors with circulating (urinary and plasma) nitrates.

**Methods:**

Using a cross-sectional design, we analyzed data collected from 5,058 adults (men and women) in the National Health and Nutrition Examination Survey (NHANES) and from 1,260 men in the Health Professionals Follow-up Study (HPFS). Urinary nitrates were measured in the NHANES, and plasma nitrates were measured in the HPFS.

**Results:**

We found an inverse association between unprocessed red meat and circulating (plasma and urinary) nitrates across two studies (NHANES and HPFS) (p for trend <0.001). Furthermore, this inverse association was stronger among participants who had reduced renal functions than participants with normal renal functions in the NHANES and stronger in men who developed prostate cancer later than men who did not develop prostate cancer in the HPFS (p<0.001 for each comparison). Moreover, African Americans had the lowest urinary nitrates among all ethnic groups. Female gender, estimated glomerular filtration rate, age, physical activity, and smoking are positively associated with urinary nitrates whereas urine albumin/creatinine ratio, body mass index, and diabetes were inversely associated with urinary nitrates. P-values are <0.05 for all of these associations. Most associations found in NHANES were similar to those found in HPFS.

**Conclusions:**

We identified new associations of red meat, and reduced renal function with circulating nitrates and confirmed the associations of other lifestyle factors with circulating nitrates identified in previous studies. Our comprehensive analyses helped identify potential confounding factors, can contribute to generating new hypotheses related to factors influencing circulating nitrates, and offer important implications for individualized nutrition.

## Introduction

The associations between circulating (plasma and urinary) nitrates and disease outcomes have been inconsistent in observational and intervention studies. Some were positively [1–6] and some were negatively associated with adverse disease outcomes. Emerging evidence from a large nested case-control study from our group and intervention studies conducted by other researchers showed inverse associations of circulating nitrates with adverse disease outcomes [7–13]. The underlying mechanism has not been well studied. Determining predictors of circulating nitrates in observational studies will help identify factors that may influence circulating nitrates, recognize potential confounding factors, and generate new hypotheses.

An emerging need exists to study predictors of circulating nitrates. Circulating nitrates are influenced not only by exogenous nitrate intakes (e.g. from vegetables) [14] but also by endogenous nitric oxides, and other endogenous factors, e.g., oral and gut microbiomes [15–18] and endogenous estrogen [19,20]. Diet and lifestyle factors and other demographic characteristics can potentially influence microbiomes [21–23], nitric oxide bioavailability [24], and estrogens [25,26]. Therefore, the changes of circulating nitrates may reflect the cumulative impact of diet and lifestyle factors on nitric oxide production, microbiome, hormone changes, nitrate intakes and other possible biological pathways.

Previous studies faced certain limitations. First, the studies examining predictors of circulating nitrates, such as renal function, did not use the commonly used biomarkers for renal function and renal damage [27], were not conducted among a general healthy population [28,29] and did not adjust for other covariates [6]. Renal disease is asymptomatic, and more than 90% of chronic kidney disease (CKD) patients are not aware of their disease status until later stages [30], when few treatment options are available. Second, the previous studies did not adjust for dietary factors or renal function together with other predictors, such as race, so their results may be subject to confounding issues. Last but not least, unprocessed red meat is one of the most controversial topics because its associations with chronic diseases have been inconsistent [31–37]. Thus, more studies are needed in this area.

Our group previously identified a novel association that usual (habitual) intakes of total red and processed meat were associated with reduced levels of plasma nitrates [13]; however, the traditional assumption would be a positive association. Thus, it is critical to confirm our finding in another large cross-sectional study. Furthermore, it is important to separate unprocessed and processed red meats to examine them along with white meat and total vegetable intakes with circulating nitrates.

The purpose of our study was to determine the associations of renal function, race, and the usual intakes of processed red meat, unprocessed red meat, white meat, and total vegetables as well as other demographic factors with urinary nitrates in order to compare whether the associations of different types of meat and other lifestyle factors with urinary and plasma nitrates are similar across two large cross-sectional studies: the National Health and Nutrition Examination Survey (NHANES) and the Health Professionals Follow-up Study (HPFS).

## Methods

### Study design and populations

The design of the study reported here was cross-sectional utilizing NHANES and HPFS cohorts. The NHANES program, established in the early 1960s, was designed to assess the health and nutritional status of representative adults and children in the United States each year in order to determine the prevalence and risk factors of major chronic diseases [38,39] The National Center for Health Statistics Research Ethics Review Board reviewed and approved NHANES, and all participants signed written consents in each year’s survey. De-identified data are accessible online. We used dietary, demographic, physical examination, dietary questionnaire, biomarkers measured in plasma and urine samples from the 2005–2006 NHANES dataset. After excluding missing urinary nitrate and non-detectable urinary nitrate values, our study consisted of 5,058 participants (18 to 85 years old). Data on gender, age, race, body mass index (BMI), smoking status, fasting status, physical activity, and diet were collected, and urinary nitrate and urinary creatinine were measured. Plasma creatinine and urinary albumin were also measured in NHANES.

The HPFS cohort study is an ongoing prospective cohort study of 51,529 men starting in 1986. Within the HPFS, a blood cohort was initiated between 1993 and 1995 among healthy participants (who were free of cardiovascular disease, diabetes, and cancer); this study was approved by the Institute Review Board at Harvard School of Public Health and Brigham and Women’s Hospital approved this study. Within this sub-cohort, plasma nitrates were measured among 1260 participants using their blood samples collected during 1993–1995, and dietary information was collected among these participants during 1993–1995. These participants were from a nested case-control study of prostate cancer (630 of them developed prostate cancer during follow-up but were free of cancer at the time of the blood draw). The details were described previously [13].

### Dietary assessment of red meat (processed and unprocessed red meat) and vegetables

Usual intakes of meat and vegetables were obtained through analysis of a non-quantitative food frequency questionnaire (NFFQ) in NHANES and were obtained from a semi-quantitative food frequency questionnaire (SFFQ) in the HPFS cohort.

#### Non-quantitative FFQ in NHANES:

The NFFQ in NHANES included a series of questions to assess usual dietary intake. We extracted information from several questions regarding intake of meat and vegetables. For unprocessed red meat, we gathered information on the following meat items: roast beef sandwiches, beef hamburgers, beef mixtures, other roast beef, steak, spare ribs, and pork. For processed red meat, we gathered information on the following items: luncheon ham, cold cuts, hot dogs, bacon, baked ham, and sausage. For white meat, we included canned tuna, chicken, turkey, chicken salad, and seafood such as raw and cooked fish or other seafood. The definition of our total vegetables are non-starchy vegetables. We gathered information on the following items: cooked greens, raw greens, coleslaw, sauerkraut, carrots, string beans, peas, corn, broccoli, cauliflower, mixed vegetables, onions, peppers, cucumbers, fresh tomatoes, squash, lettuce salads, salsa, catsup, pickles, and an “other vegetables” category.

#### The NFFQ assesses average frequency of consumption of each food over the past year:

Never, one to six times per year, seven to eleven times per year, one time per month, two to three times per month, one time per week, two times per week, three to four times per week, five to six times per week, one time per day, and two or more times a day. For this study, we converted the above-mentioned frequencies into daily frequencies. For example, responses recorded as “three to four times per week” were given a value of 0.5 (3.5 times/7 days=0.5 times per day). We calculated the daily frequency for vegetables and processed and unprocessed red meat using the NFFQ by summing the daily frequencies of our selected foods.

#### Semi-quantitative FFQ (SFFQ) in HPFS:

Beginning in 1986, in the HPFS, the SFFQ was sent to participants every 4 years to update information on their diet. The reproducibility and validity of these SFFQs in measuring food intake have been described previously in detail [40–43]. We used the information from the 1994 SFFQ, which asked participants to report their usual intakes (never to ≥ 6 times/d) of a standard portion size (e.g. 4–6 oz of steak). Frequencies and portions for the individual food items were converted to average daily intake of each food item for each participant. Processed red meat comprising sausage, salami, bologna, bacon, and hot dogs, and average daily intakes of individual processed red meat items were summed to compute total processed red meat intakes. Unprocessed red meat comprising pork, beef or lamb, calf in main dishes, hamburger or sandwiches, pork liver, and chicken or turkey liver and sum of unprocessed red meat was computed. For white meat, we included chicken, turkey, chicken or turkey dogs, canned tuna, and any fish or shrimp. Our non-starchy total vegetable intakes included tomatoes (including tomato juice and sauce), string beans, broccoli, cabbage, cauliflower, Brussels sprouts, carrots (raw and cooked), corn, lima beans, beans or lentils, squash, eggplant, zucchini or other squash, spinach, kale, chard greens, lettuce, celery, green peppers, onions, and mixed vegetables. We did not include potatoes or soy in our definition of vegetables.

Intakes of red meat, white meat and vegetable from SFFQ were validated previously using two 1-week diet records. Pearson correlations between SFFQ and diet records were 0.49–0.5 for intakes of red meat (processed and unprocessed) and white meat, and 0.3–0.5 for intakes of vegetable [44].

### Estimated Glomerular Filtration Rate (eGFR) and urinary albumin/creatinine (ACR) in the NHANES

NHANES has measured serum creatinine, which allow us to calculate eGFR, the most commonly used method to estimate renal function. Serum creatinine was measured via Jaffe rate method using Beckman Synchron LX20 analyzer [45]. We calculated eGFR using CKD-EPI equation [46]. We categorized eGFR into the following clinically relevant categories: ≥ 90, 60–89, and <60 ml/min/1.73 m^2^. Urinary albumin level was measured by solid-phase fluorescence immunoassay, and urinary creatinine level was measured by the modified kinetic method of Jaffe using a Beckman Coulter Synchron AS/Astra Analyzer. Albuminuria was expressed as urinary albumin-to-creatinine ratio (ACR) and categorized into 2 categories; <30 mg/g, and ≥ 30 mg/g.

### Urinary and tap water nitrate assessment in the NHANES

One spot morning urine sample was collected from each participant at the initial Mobile Examination Center interview in the NHANES study and urine was not collected in HPFS cohort. Urine samples were sent to a lab to be processed and analyzed for various analytes, including urinary nitrates. The method for analyzing urinary nitrates in this study was described previously (http://wwwn.cdc.gov/nchs/nhanes/2005-2006/PERNT_D.htm). Briefly, urinary nitrates were measured using ion chromatography coupled with electrospray tandem mass spectrometry. We used both urinary nitrate values (ng/ml) and creatinine (mg/dl) from each participant to calculate a creatinine-adjusted urinary nitrate value (ng/mg). Adjusted urinary nitrate with creatinine allows for the adjustment of urine concentrations (i.e., nitrate values will not be influenced by concentrated or diluted urines). In addition, nitrate concentration in tap water was measured in a subset of 1301 participants in the NHANES using the same method.

### Assessment of physical activities

We extracted data from self-reported leisure time physical activities. Metabolic equivalent task (MET) were calculated based on frequencies and durations of each activity using activity code in the 2011 Compendium of Physical activities, a valid used instrument to quantify the energy expenditure of physical activity [47].

### Plasma nitrate assessment in the HPFS

Plasma samples were collected in HPFS cohort. Measuring plasma nitrates in the HPFS cohort followed Griess’s method using colorimetric detection. The details were described previously [13].

### Statistical analysis

All statistical analyses were conducted in SAS 9.3 (SAS Institute, Cary NC). For NHANES data, urinary nitrate/creatinine was not normally distributed and thus was log transformed to better meet model assumptions. We used linear regression models with log transformed urinary nitrate/creatinine as the dependent variable. In multivariable models, we adjusted for age, gender, race, and other potential covariates, which have also been found to be associated with nitrate levels. These variables include BMI, smoking status, fasting status, and physical activity. In addition, because NHANES is a survey, weights are created in NHANES to account for the complex survey design (including oversampling), survey non-response, and post-stratification. When a sample is weighted in NHANES, it is representative of the U.S. Census civilian non-institutionalized population. Therefore, we also included the 2-year sample weights and design factors for these analyses, as appropriate (full sample 2-year interview weight and full sample 2-year Mobile Examination Center exam weight). In addition, in the subset of 1301 participants who had nitrate measures in the drinking tap water, we calculated the amount of nitrates from tap water intake/day which was = nitrate concentration in water × total amount of tap water intake/day. We then analyzed the associations between nitrate intake from tap water and urinary nitrate in the multivariate-adjusted model.

For HPFS data, to analyze plasma nitrate levels in relation to food intakes, we used multivariable linear regression analyses. Plasma nitrate was treated as a dependent variable and was log transformed for the same reasons as urinary nitrate/creatinine. The regression model was adjusted for age, BMI, smoking status, fasting status, and physical activity.

Finally, we analyzed the joint association of red meat with another variable with circulating nitrates. In the NHANES, to determine the joint association of unprocessed red meat plus renal function with urinary nitrates, we created four groups (e.g. high GFR and low red, high GFR and high red meat). We used the quintile 4 as the cut-off point for red meat to separate high and low red meat groups. To remove the confounding by vegetables, we elected to determine the association among those who had high intakes of total vegetables (above the median in the NHANES). Similarly, in the HPFS, we created four groups based on red meat and advanced prostate cancer status. Although our analyses were cross-sectional, we did have information on who later developed advanced prostate cancer (clinical stage ≥ 2c) after blood draw in the cohort. To assess whether the combination of red meat and other joint variable had any synergistic association with circulating nitrates, we used the Wald P-value for the interaction term in a model that also included the main effects.

## Results

### Baseline characteristics of NHANES and HPFS studies

Table 1 provides demographic and health characteristics of the NHANES participants, of whom approximately 52% were women, 45% were over 45 years old, 25% were African American, more than 60% were overweight, 22% were current smokers and 25% were past smokers, 39% had sedentary and 16% had active physical lifestyles, more than 40% had elevated blood pressure or hypertension at/above stage 1, more than 10% had diagnosed or borderline diabetes, and approximately 35% had renal insufficiency (eGFR of 60–90 ml/min/1.73m^2^) while 8% had moderate reduced renal function (eGFR<60 ml/min/1.73m^2^). Nearly 12% of participants had microalbuminuria (urinary ACR>30mg/g). Approximately 50% of participants provided postprandial urine samples (<8 hr after last meal; data not shown). The details regarding characteristics in the HPFS cohort were previously described [13]. Briefly, they were all men aged 47 to 81 years; 94% were white, and 50% were overweight. Only 4% of men were current smokers in the HPFS cohort.

### Processed and unprocessed red meat, white meat, and total vegetable intakes and circulating nitrate levels in NHANES and HPFS

Urinary nitrates were measured in NHANES, and plasma nitrates were measured in HPFS. To examine the association of processed and unprocessed red meat, white meat, and vegetables with circulating nitrates, we simultaneously adjusted processed and unprocessed red meat, white meat, and vegetables after controlling for the covariates listed in the footnote of Table 2. We found positive associations between total vegetable intakes and circulating nitrates in both NHAES and HPFS studies. Because the dependent variables (urinary or plasma nitrates) were log transformed, the percent difference was mathematically equivalent to the beta estimate. Using quintile 1 as the reference group, the percent differences between extreme quintiles of total vegetables were 25% (p=0.0001) in NHANES and 17% (p=0.0002) in HPFS. For different types of meat, in the NHANES study, we found that both processed and unprocessed red meats were inversely associated with urinary nitrates; however, the percent differences between extreme quintiles were −6% for processed and −15% for unprocessed red meat, while p for trend was only significant for unprocessed red meat (p=0.001). For HPFS, the inverse associations between processed and unprocessed red meat and plasma nitrates were both statistically significant; however, the association was stronger for processed red meat. The percent differences between extreme quintiles were −11% for processed red meat (p=0.0001) and −8% for unprocessed meat (p=0.05). White meat was not associated with urinary nitrates in NHANES or with plasma nitrates in HPFS.

### Associations between renal function and urinary nitrates in the NHANES

In this study, we found that renal function and kidney damage were inversely associated with urinary nitrates. In Table 3, compared to participants with normal renal function (eGFR>90), those with mild reduced (eGFR 60–90) and moderate reduced renal function (eGFR<60) were associated with a 14% (p<0.0001) and 40% (p<0.0001) reduction of urinary nitrates, respectively. Participants with kidney damage (ACR ≥ 30 mg/g) had an 8% (p=0.008) reduction of urinary nitrates compared to those with ACR<30 mg/g.

We further examined the joint associations of eGFR and ACR with urinary nitrates. Participants with both kidney damage (ACR ≥ 30) and moderate reduced renal function (GFR<60) had the lowest urinary nitrates with a 59% reduction of urinary nitrates (p<0.0001) compared to participants with GFR>90 and ACR<10 (the reference group). Compared to the reference group, we observed reductions even among those with a normal ACR ratio (ACR of 10–29.9) and participants with renal insufficiency (GFR of 60–90); the magnitudes of the inverse associations were stronger among higher ACR and lower eGFR groups (Table 4).

### Other demographic and lifestyle factors with urinary nitrates in NHANES

We further examined other factors associated with urinary nitrates besides diet. African Americans had the lowest levels of urinary nitrates — namely, 28% lower (p<0.0001) compared to whites. In addition, female gender, age, smoking status, and physical activity were positively associated with urinary nitrates (p<0.05 for all of these associations). Diabetes status, BMI, and fasting hours were inversely associated with urinary nitrates (p<0.05 for all of these associations). Women had 20% higher urinary nitrates than men; participants over 45 years old had 24% higher urinary nitrates than younger participants (ages 18–30); current smokers had 20% higher urinary nitrates than never smokers; and those who engaged in rigorous activities (≥ 1200 METs min/week) had 9% higher levels of urinary nitrates than those engaging in less rigorous activities (<600 METs min/week). Furthermore, those who were obese had 20% lower urinary nitrates than individuals who were of normal weight; participants with diabetes had 9% lower urinary nitrates than normal individuals and individuals who had fasted for more than 8 hours had 7%–8% lower urinary nitrates than those who did not provide fasting urine samples (Table 5).

### Joint associations of red meat and renal function with urinary nitrates and joint association of red meat and incident advanced prostate cancer with plasma nitrates

Compared to the reference group (low red meat intakes plus eGFR ≥ 60), the reduction of urinary nitrates was 32% (p=0.003) among those with eGFR<60 and high red meat intakes but only 10% (p=0.01) among those with eGFR ≥ 60 and high red meat intakes. P-values for interaction were <0.05. Similarly, compared to healthy men with low red meat intakes, the reduction of plasma nitrates was 45% (p<0.001) in men with prostate cancer and high red meat intakes whereas the reduction was only 9% (p=0.007) in healthy controls with high red meat intakes. Although the p for interaction was marginally significant (p=0.09), the p-value for point-estimates were significant.

### Amount of nitrate in tap water in relation to urinary nitrate

In our multivariate analyses among 1301 participants, we found that the amount of total nitrate intake from tap water was positively associated with urinary nitrates (data are not shown). Compared to those in the bottom quintile of nitrate intake (0 g nitrate/day) from tap water, those in the top quintile of nitrate intake (14 g nitrate/day) showed a 15% increase in urinary nitrates (P for trend was <0.0001). In this subset of samples, estimates for other predictors did not change materially with and without the adjustment of tap water in the multivariate model.

## Discussion

Our study is the first to provide evidence of the inverse associations between processed and unprocessed red meat and circulating nitrates; the strengths of the inverse associations between red meat and circulating nitrates differ by incident prostate cancer status and renal function and have important implications. Our study extended the previous results on creatinine and urinary nitrates and demonstrated that the reduced renal function was associated with reduced urinary nitrates using the two most commonly clinical markers (eGFR and ACR). We further confirmed associations identified from previous studies that age, female gender, physical activity, and current smoking are positively associated with urinary nitrates [13,48], whereas BMI was inversely associated with urinary nitrates [13] and African Americans had the lowest levels of urinary nitrates among all ethnic groups [49].

Our cross-sectional study demonstrated that low urinary nitrates were associated with reduced renal function (eGFR) and kidney damage (ACR) in the general healthy population not diagnosed as CKD. Urinary nitrates are sensitive to reduced renal function, as we found a 14% reduction of urinary nitrates even among participants with eGFR of 60–90 compared to those with GFR ≥ 90. The significant joint associations of eGFR and ACR with urinary nitrates further confirmed that reduced renal function and increased renal damage were associated with lower urinary nitrates. Previous cross-sectional studies found an inverse association between serum creatinine and urinary or plasma nitrates, and circulating nitrates were lower in patients with chronic CKD patients as compared to healthy controls [28,29]; however, the two commonly used clinical markers (eGFR and ACR) were not measured [27–29]. Further these studies focused on patients at an intensive therapy unit [27], with CKD [28] or end-stage CKD patients [29] but not among the general population and the sample size of the studies were small. Although one study showed high eGFR was associated with low plasma nitrates, the magnitude was negligible and diet, race and other covariates were not adjusted in the model [6]. Moreover, for the first time we demonstrated that the inverse associations were independent of hypertension and diabetes and not mediated by hypertension and diabetes because the adjustment with/without these two factors did not materially change the associations.

Notably, our results provide strong evidence that low circulating nitrates are also associated with adverse outcomes—namely, reduced renal function and increased kidney damage. Our results, together with findings linking low nitrates with aggressive prostate cancer in our previous nested case-control study [13] as well as increased circulating nitrates associated with improving cardiovascular outcomes in intervention studies [8–12] indicate that low circulating nitrates are linked to adverse outcomes. The traditional assumption is that high levels of circulating nitrates are harmful because they are associated with several types of cancer [1–5] and total mortality [6]. Summarizing the associations of high- and low-nitrates with adverse outcomes suggests a potentially U-shaped relationship between circulating nitrates and human health.

Ours was the first study to demonstrate inverse associations between processed and unprocessed red meat and circulating nitrates while white meat was not associated with circulating nitrates in either NHANES or HPFS studies. The differential pattern for red and white meat with circulating nitrates suggests that some components existing only in red meat may influence the association. For instance, heme can promote the formation of N-nitroso compounds (e.g., nitrosamines) [50]. Red and white meats influence microbiomes differently [51–53]. All of these may influence nitrate metabolism, further influencing circulating nitrate levels. More mechanistic studies are needed. The stronger inverse associations between red meat and circulating nitrates in men with advanced prostate cancer or participants with reduced renal function provide important evidence of individualized nutrition. Although our results are subgroup analyses and require further confirmation, this finding has important implications as the WHO’s classification of unprocessed red meat as Group 2 carcinogens has stirred up huge debates in research communities. The evidence linking red meat and cancer (other than colorectal cancer) is inconsistent [31–37]. Our results suggest that the inconsistent results may partially depend on their circulating nitrate levels, renal functions, and susceptibility to prostate cancer. If our results are supported by other studies, circulating nitrates may be used as a tool to screen individuals susceptible to red meat intakes.

Our study confirmed most of the associations of the lifestyle factors with circulating nitrates in previous studies. These consistencies support the validity of our study. Of note, we confirmed that females had higher urinary nitrates than males [48,49]. Animal studies have shown that nitrates can inhibit androgen production [54,55] and estrogen itself can increase circulating nitrates [19,20,56]; however, whether this is one mechanism for women regulating estrogen–androgen balance merits further investigation. Our findings on the positive associations of total vegetable intakes, age, smoking, physical activity, and the inverse association of BMI with urinary nitrates in NHANES were consistent with the associations of these variables with plasma nitrates in our previous cross-sectional analyses in HPFS [13] but HPFS included only men. The fact that vegetable intakes increase circulating nitrates has been demonstrated in many intervention studies [57–59]. The positive association between current smoking and urinary nitrates is in contrast with another cross-sectional study, Jain in 2016, which reported a negative association [49]. The association between smoking and circulating nitrates is complex. Smoking causes oxidative stress and can upregulate nitric oxide production by stimulating inducible nitric oxide synthase [60,61]; on the other hand, smoking can also inhibit nitric oxide production and bioavailability by inhibiting endothelial nitric oxide synthase and endothelial cell function [62,63]. Therefore, the cumulative impact of smoking on circulating nitrates merits further investigation in longitudinal studies. Moreover, we found an inverse association between diabetes and urinary nitrates, although the associations with urinary nitrates and diabetes or insulin resistance were not consistent [64–66]. Whether this is due to the use of diabetic medications and whether diabetes is well controlled will need to be determined in the future. Finally, we confirmed results from other studies that African Americans had the lowest levels of urinary nitrates among all ethnic groups [49]. The lowest levels of urinary nitrates in African American indirectly support our previous results that low plasma nitrates were associated with aggressive prostate cancer [13], as African Americans have the highest prevalence of advanced-stage prostate cancer [67] and the highest incidence and death rate of prostate cancer among all ethnic groups [68–70].

Our study has several strengths. First, NFFQ and SFFQ both assess usual dietary intakes. Our study is the first large study to assess the association of usual intakes of vegetables, white meat, and unprocessed and processed red meat simultaneously with circulating nitrates. Second, this study covered a heterogeneous group including both men and women; Whites, African Americans, and other racial ethnic groups; and a wide age range (from 18 to 81). Third, if our results are confirmed in longitudinal studies and intervention studies, our results will also have implications for future nitrate-supplement interventions. Knowing which foods and lifestyle factors may potentially lower circulating nitrates will help investigators decide which foods to avoid and which population to study in order to improve the efficiency of the intervention.

The study has several limitations. First, our cross-sectional design cannot provide causal associations. However, this is often the first step in generating hypotheses. Second, observational studies are subject to confounding factors; however, we have adjusted for multiple potential confounding factors and examining multiple predictors simultaneously. The consistent results from NHANES and HPFS help validate each other. Third, FFQ is designed to capture the frequency of usual intakes not exact intakes, and the exact amount of intakes of red meat with circulating nitrates will need to be determined in the future. Fourth, the HPFS cohort only included men and did not measure serum creatinine or urine albumin; furthermore, it comprises mainly Whites. Thus, the associations of renal function, race, and gender with plasma nitrates cannot be measured. However, the results did not change materially in NHANES even when we removed the variables of renal function, race, and gender. Nitrates in tap water were only measured in a subset of NHANES; however, the results in the whole data set should not be influenced by the nitrates in tap water as we did not find significant changes with/without the adjustment of tap water in the subset of samples. Fifth, we do not have information on the use of anti-bacterial mouth rinses, which can influence the oral microbiomes and potentially influence circulating nitrates. However, the use of such mouth rinses would unlikely be associated with all the predictors, and we observed consistent results across two studies, thereby suggesting that, if any influence stemmed from this factor, it would likely result in random errors, leading the associations toward null. Finally, in such a large human study, we cannot study nitrate pharmacodynamics (i.e., the enterosalivary circulation of nitrates) as studies focusing on pharmacodynamics are usually small in scale; however, our study complements those studies.

## Conclusion

In summary, our study confirmed some existing and identified some new associations of dietary and lifestyle factors with circulating nitrates. Our subgroup analyses of red meat and circulating nitrates provide important data to study individualized nutrition, which is highly promoted by National Institute of Health and several other health organizations. We currently lack of specific guidelines for red meat intake for subgroups. Our results help identify confounding factors that may influence circulating nitrates and facilitate the summarization of the comprehensive relationship of circulating nitrates with chronic diseases in the future.

## Supplementary Material

Suppl

## Figures and Tables

**Table 1:** Baseline characteristics of study population the National Health and Nutritional Examination Survey.

Variables	N (Percent)	Median (inter-quartile range)-urinary nitrate, µg/mg (creatinine adjusted)
Cr.-adj urinary nitrate, ng/mg	5071 (100)	38.4 (27.4, 55.2)
**Gender**		
Men	2446 (48.3)	35.3 (25.9, 50)
Women	2625 (51.7)	41.3 (29.2, 60.6)
**Age, years**		
Ages 18–30	1590 (31.3)	34.0 (26.6, 45.1)
Ages 31–45	1192 (23.5)	38.7 (28.3, 53.9)
Ages 46–65	1326 (26.2)	38.3 (28.0, 53.9)
Ages >65	963 (18.9)	41.6 (29.2, 58.7)
**Race**		
Non-Hispanic White	2373 (46.8)	42.0 (29.8, 59.0)
Mexican American	1090 (21.5)	41.7 (30.1, 58.1)
Other Hispanic	160 (3.2)	37.5 (26.4, 52.7)
Non-Hispanic Black	1239 (24.5)	29.6 (22.2, 40.6)
Other race	209 (4.0)	43.3 (34.0, 66.8)
**Body mass index (BMI), kg/m** ^2^		
BMI<18.5	92 (1.8)	46.9 (29.3, 67.3)
18.5 ≤ BMI <25	1549 (30.9)	40.4 (28.7, 57.6)
25 ≤ BMI <30	1662 (33.1)	38.6 (28.0, 55.4)
BMI ≥ 30	1713 (34.2)	36.2 (26.0, 2)
**Smoking Status**		
Never	2400 (52.9)	37.4 (26.6, 54.6)
Past	1152 (25.4)	37.1 (26.1, 55.2)
Current	983 (21.7)	44.6 (32.9, 60.8)
Physical Activity, MET*min/wk		
Low (<600)	1238 (38.6)	39.0 (27.8, 55.7)
Moderate (600–1199)	1446 (45.1)	38.4 (27.9, 56.5)
High (≥ 1200)	524 (16.3)	37.5 (26.8, 51.8)
**Blood Pressure**		
Normal	1629 (46.5)	39.4 (28.7, 55.7)
Elevated	644 (12.7)	37.3 (26.5, 53.1)
Hypertensive	1711 (33.7)	37.6 (26.1, 54.2)
missing	356 (7.1)	36.5 (25.8, 54.1)
**Diabetes status**		
No	4532 (89.5)	38.5 (27.7, 54.9)
Yes	453 (8.9)	36.1 (22.2, 56.7)
Borderline	81 (1.6)	41.6 (28.9, 60.9)
**eGFR, mL/min/1.73 m** ^2^		
>90	2773 (57.6)	39.6 (29.1, 55.9)
60–90	1672 (34.8)	38.1 (26.5, 55.2)
<60	366 (7.6)	27.7 (18.9, 44.9)
**ACR, mg/g**		
<30	4458 (88.1)	38.8 (27.9, 55.3)
≥ 30	604 (11.9)	35.2 (23.5, 53.9)

NOTE: ACR=albumin-creatinine ratio, eGFR=estimated glomerular filtration rate, MET=metabolic equivalent

**Table 2:** The associations between urinary nitrates and food intakes in the NHANES and plasma nitrates and food intakes in the HPFS.

Urinary nitrates and quintiles of food intakes							
NHANES study		Quintile 1	Quintile 2	Quintile 3	Quintile 4	Quintile 5	P for trend
Processed red meat	Median (times/d)	0.06	0.18	0.32	0.55	1.1	
	Beta (p-value)	ref	−0.05 (0.5)	−0.04(0.4)	−0.03(0.1)	−0.06 (0.06)	0.2
Unprocessed red meat	Median (times/d)	0.1	0.27	0.45	0.7	1.25	
	Beta (p-value)	ref	−0.11(0.1)	−0.14(0.05)	−0.12 (0.03)	−0.15 (0.001)	0.007
White meat	Median (times/d)	0.09	0.2	0.35	0.6	1.11	
	Beta (p-value)	ref	0.03 (0.9)	0.02 (0.8)	0.02 (0.7)	0.02 (0.8)	0.9
Vegetables	Median (times/d)	0.74	1.78	2.85	4.2	6.6	
	Beta (p-value)	ref	0.13 (0.01)	0.14 (0.001)	0.11 (0.0001)	0.23 (0.0001)	<0.0001
Plasma nitrates and quintiles of food intakes							
HPFS Study		Quintile 1	Quintile 2	Quintile 3	Quintile 4	Quintile 5	P for trend
Processed red meat	Median (serving/d)	0	0.08	0.16	0.3	0.65	
	Beta (p-value)	ref	−0.05 (0.05)	−0.09 (0.002)	−0.09 (0.001)	−0.11 (0.0001)	0.0006
Unprocessed red meat	Median (serving/d)	0.19	0.39	0.53	0.76	1.19	
	Beta (p-value)	ref	−0.05 (0.07)	−0.06 (0.06)	−0.08 (0.03)	−0.08 (0.05)	0.05
White meat	Median (serving/d)	0.3	0.5	0.67	0.87	1.23	
	Beta (p-value)	ref	−0.01 (0.8)	−0.05 (0.9)	0.03 (0.8)	0.001 (0.8)	0.9
Vegetables	Median (serving/d)	1.79	2.63	3.44	4.62	6.55	
	Beta (p-value)	ref	0.07 (0.01)	0.14 (0.03)	0.09 (0.01)	0.17 (0.0002)	<0.0001

Covariate adjusted in the NHANES study: age, gender, race, body mass index, smoking status, physical activity, and fasting status and glomerular filtration rate.

Covariate adjusted in the HPFS study: age, race, body mass index, smoking status, physical activity, and fasting status.

Processed red meat, unprocessed red meat, white meat and vegetables were adjusted simultaneously in the model. NHANES denotes the National Health and Nutritional Examination Survey; HPFS denote Health Professionals Follow-up Study.

**Table 3:** The associations between renal function and log creatinine-adjusted urinary nitrate levels (ng nitrate/mg creatinine) in the National Health and Nutritional Examination Survey.

Renal function	β estimate (P value)
**eGFR, mL/min/1.73 m** ^2^	
>90	Ref
60–90	−0.14 (<0.0001)
<60	−0.40 (<0.0001)
**ACR, mg/g**	
<30	Ref
≥ 30	−0.08 (0.008)

NOTE: eGFR=estimated glomerular filtration rate. ACR=albumin-creatinine ratio. eGFR and ACR were entered into the model separately.

Covariates include gender, age, race, body mass index, smoking status, physical activity, diabetes, intakes of red meat (processed plus unprocessed red meat) and total vegetables and fasting status.

GFR and ACR were not adjusted simultaneously

**Table 4:** Joint associations of eGFR and ACR with urinary nitrates in the in the National Health and Nutritional Examination Survey.

	ACR <10	ACR 10–29.9	ACR ≥ 30
	β estimate (P value)	β estimate (P value)	β estimate (P value)
eGFR>90	Ref	−0.0033 (0.91)	0.047 (0.29)
eGFR 60–90	−0.13 (<0.0001)	−0.15 (<0.0001)	−0.17 (0.0004)
eGFR<60	−0.30 (<0.0001)	−0.37 (<0.0001)	−0.59 (<0.0001)

NOTE: eGFR=estimated glomerular filtration rate; ACR=albumin-creatinine ratio; eGFR and ACR combination variable were entered in to the model adjusting all covariates.

Covariates include gender, age, race, body mass index, smoking status, physical activity, diabetes, intakes of red meat (processed plus unprocessed red meat) and total vegetables and fasting status.

**Table 5:** Multivariable-adjusted associations between non-dietary and lifestyle factors in 2005–2006 and log transformed urinary nitrate levels (ng nitrate/mg creatinine) among study participants in the National Health and Nutritional Examination Survey.

Non-dietary and lifestyle factors in 2005–2006		β estimate (P value)
Gender	Men	Ref
	Women	0.20 (<0.0001)
Age, y	18 ≤ age <30	Ref
	30 ≤ age <45	0.076 (0.0706)
	45 ≤ age <65	0.235 (<0.0001)
	AGE ≥ 65	0.242 (<0.0001)
Race	Non-Hispanic White	Ref
	Mexican American	0.01 (0.07)
	Other Hispanic	−0.10 (0.025)
	Non-Hispanic Black	−0.28 (<0.0001)
	Other Race - Including Multi-Racial	0.12 (<0.0001)
BMI, kg/m2	BMI <18.5	−0.09 (0.1)
	18.5 ≤ BMI <25	ref
	25 ≤ BMI <30	−0.08 (0.5)
	BMI ≥ 30	−0.20 (<0.0001)
Smoking Status	Never	Ref
	Past	0.06 (0.005)
	Current	0.20 (<0.0001)
Physical Activity, MET* min/WK	Low (<600)	Ref
	Moderate (600–1199)	0.05(0.2)
	Rigorous (≥1200)	0.09 (0.0004)
Diabetes status	No	Ref
	Borderline	0.09 (0.2)
	Yes	−0.09 (0.006)
Fasting, hr	≤ 8 hours	Ref
	8< hours ≤ 12	−0.08 (<0.0001)
	12< hours	−0.07(<0.004)

Covariates include the all the exposure variables (gender, age, race, BMI, smoking status, physical activity, diabetes status and fasting), as well as intakes of unprocessed red meat, processed red meat, and vegetables, and eGFR. eGFR=glomerular filtration rate; BMI=body mass index; MET=metabolic equivalent
