# Supplementary material for: Red Meat, Renal Function, Race and Other Predictors of Circulating Nitrates
Source: Epidemiology (Sunnyvale). Author manuscript; Available in PMC 2025 Oct 29. (PMC12560619)
Supplement: Suppl [file NIHMS1030132-supplement-Suppl.pdf]

**NHALES**	**L-red meat Beta-estimate(p-value)**	**H-red meat Beta-estimate(p-value)**
eGFR ≥ 60(n=1500)	Ref	-0.10(p=0.009)
eGFR<60(n=240)	-0.15(p=0.006)	-0.32(p<0.001)
Covariates include gender, age, race, body mass index, smoking status, physical activity, diabetes, and fasting status. eGFR denotes glomerular filtration rate; NHANES denotes the National Health and Nutritional Examination Survey. L-meat denotes low red meat intakes; H-red meat denotes high red meat intakes. Those have red meat intakes ≥quintile 4 in the NHANES are considered as having high intakes.		

Table S1: Joint association of red meat and renal function with urinary nitrates among participants who had intakes of total vegetables above the median in the NHANES.

**HPFS**	**L-red meat Beta-estimate(p-value)**	**H-red meat Beta-estimate(p-value)**
Controls (n=155)	Ref	-0.16(p=0.009)
Advanced PCa (n=30)	-0.15(p=0.006)	-0.49(p<0.001)
Covariates include age, race, body mass index, smoking status, physical activity, diabetes, and fasting status. PCa denotes prostate cancer; HPFS denote Health Professionals Follow-up Study. L-meat denotes low red meat intakes; H-red meat denotes high red meat intakes. Those have red meat intakes ≥ quintiles 4 in the HPFS are considered as having high intakes.		

Table S2: Joint association of red meat and advanced PCa* status with plasma nitrates among men who had intakes total vegetables above the median in the HPFS cohort.
